# High-Precision Localization Algorithm for Target Symmetry Center in Image-Based Overlay Metrology

**DOI:** 10.3390/mi17050626

**Published:** 2026-05-20

**Authors:** Wuhao Liu, Maoxin Song, Shuming Shi, Mingchun Ling, Hengwei Qin, Hengrui Guan, Jun Wang, Jin Hong

**Affiliations:** 1 School of Environmental Science and Optoelectronic Technology, University of Science and Technology of China, Hefei 230026, China; liuwuhao@ustc.edu (W.L.);; 2 Anhui Institute of Optics and Fine Mechanics, Hefei Institutes of Physical Science, Chinese Academy of Sciences, Hefei 230031, China

**Keywords:** overlay metrology, center localization, correlation

## Abstract

Achieving high-precision overlay target center localization is critical for image-based overlay (IBO) metrology in advanced semiconductor manufacturing. This paper proposes a novel IBO target localization algorithm based on symmetry center matching. Leveraging the symmetry design of the IBO optical system as a physical prior, the algorithm reformulates center localization as a global correlation optimization problem. The grayscale projection profile of a single-sided edge is extracted, spatially mirrored, and used as a reference template for sliding correlation matching against the opposite edge. The symmetry center is then determined from the peak of the Pearson correlation coefficient curve. Simulation results demonstrate a center localization accuracy better than 0.00013 pixels (3σ), with repeatability precision remaining within 0.012 pixels (3σ) under stringent noise and blur conditions. Experimental validation yields object-space repeatability precision of 0.129 nm (3σ) and 0.144 nm (3σ) in the X and Y directions, respectively, surpassing the 0.32 nm measurement uncertainty requirement for advanced process nodes. The average single-frame processing time is approximately 0.07 s, demonstrating that the proposed algorithm simultaneously satisfies the demands of high precision and high throughput.

## 1. Introduction

In semiconductor manufacturing, critical dimension (CD) control and overlay (OVL) precision have consistently served as the core criteria for evaluating lithographic quality. OVL precision characterizes the alignment offset that occurs when a mask pattern is stacked onto existing structures on a wafer [1]. If this offset exceeds the designed tolerance budget, the device morphology will deviate significantly from the intended model, potentially inducing critical electrical defects such as short circuits or open circuits [2,3]. Therefore, stringent OVL control is the cornerstone for ensuring high chip yield and consistency of electrical performance.

According to the evolutionary roadmap projected by the 2024 International Roadmap for Devices and Systems (IRDS), OVL tolerance is expected to tighten to approximately 1.6 nm around 2028 [4]. Based on the industrial standard that measurement uncertainty should be kept within 20% of the tolerance budget, OVL metrology systems are required to maintain sufficient accuracy, repeatability, and throughput at an uncertainty level of approximately 0.32 nm. In recent years, with the rapid development of process scenarios such as High-NA EUV, advanced DRAM, 3D NAND, wafer bonding, and heterogeneous integration, OVL control is no longer confined to conventional global inter-layer registration, but has progressively expanded to more complex metrology targets including higher-order intra-field distortion, on-product overlay (OPO), after-develop inspection (ADI)/after-etch inspection (AEI) discrepancy, non-zero offset (NZO) stability, and in-device overlay [5,6,7,8]. For instance, research on high-volume manufacturing control for advanced devices has further indicated that achieving sub-2 nm OPO targets demands higher-density OVL sampling, faster move-and-measure capability, and more robust metrology recipes [6]. Collectively, these studies suggest that the OVL metrology error budget in advanced process nodes is rapidly approaching the performance boundary of conventional optical metrology methods.

**Figure 1:**
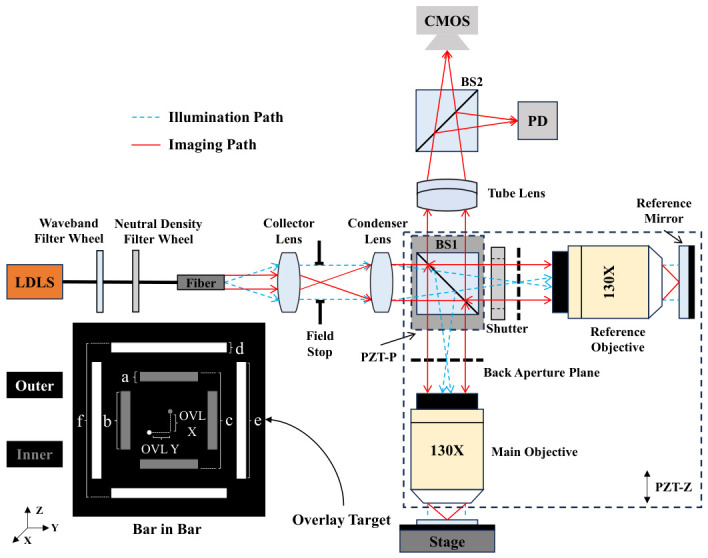
Principles of IBO optical system.

Image-based overlay (IBO) is one of the mainstream OVL metrology technologies currently in use [9,10]. Its optical measurement system consists of three subsystems: an illumination subsystem, a microscopic imaging subsystem, and an interferometric focusing subsystem. The measurement principle is illustrated in Figure 1, which is similar to the schematic diagram in our previous work [11]. The illumination subsystem employs a Köhler illumination scheme, with the illumination optical path indicated by the blue dashed lines in the figure. A Laser-Driven Light Source (LDLS) is selected as the illumination source owing to its advantages of high brightness, broad spectral range, and high stability. A filter wheel and a neutral-density filter wheel are incorporated into the optical path, enabling free selection of the illumination wavelength band and intensity. The optical path of the microscopic imaging subsystem is indicated by the red solid lines in the figure. The objective lens is designed with low aberration and object-space telecentricity, and the imaging signal is captured by a CMOS camera. The interferometric focusing subsystem employs Linnik scanning white light interferometry. When the subsystem is operating, the shutter in the reference optical path is opened, and a piezoelectric ceramic transducer (PZT) motor PZT-Z drives the interference module within the black dashed box in Figure 1 to step rapidly along the Z direction. The interference signal is received by a photodiode detector (PD). By synchronously acquiring the PZT-Z position and PD interference signal, the PZT-Z position corresponding to the focal plane can be determined. The autofocus strategy follows our previous work [12].

In Figure 1, the white rectangular blocks of the measured OVL target are designated as “outer,” and the gray rectangular blocks are designated as “inner,” where outer and inner correspond to different layers on the wafer. The thickness between layers is determined by the fabrication process of different production lines, and can often reach the order of hundreds of nanometers or even micrometers. The OVL is defined as the misregistration (MIS) error between the center positions of outer and inner targets in both the X and Y directions. In Figure 1, “OVL X” and “OVL Y” are labeled, where the gray dot represents center of inner and the white dot represents center of outer.

Research aimed at improving IBO metrology performance has in recent years focused primarily on three aspects: target structure optimization, metrology recipe control, and imaging quality enhancement. In terms of target structure, small-pitch AIM, rAIM, and more compact dense IBO targets have been employed to increase the density of measurable alignment pairs per unit area and reduce target-to-device bias and NZO variation, thereby enhancing the stability of OPO metrology [13,14,15,16]. In terms of metrology recipe, selecting appropriate illumination wavelengths and focus positions for different process layers, combined with recipe optimization using evaluation metrics such as center of symmetry, can further improve OVL metrology precision [17,18,19]. In terms of imaging quality, focus offset, target signal-to-noise ratio, and target defects directly affect the extraction accuracy of the outer and inner center positions; accordingly, several studies have proposed methods such as focus control, dual-wavelength/dual-detector imaging, and deep learning to improve image stability and metrology efficiency [20,21]. These studies collectively indicate that the fundamental objective of target structure design, recipe optimization, and imaging quality improvement is to obtain more stable, well-defined outer and inner image features that more faithfully reflect the true geometric relationships. Since OVL results are ultimately determined by the difference between the outer and inner center coordinates, further improving the accuracy, robustness, and computational efficiency of the target center localization algorithm, on top of existing IBO imaging systems, represents a critical step toward overcoming the IBO metrology performance bottleneck at advanced process nodes.

Currently, target center localization technology primarily relies on two approaches: edge fitting and template matching. The edge fitting method detects sub-pixel contour edges using the moment method and partial differential method, and then performs localization by fitting the contour center and edge information [22,23,24,25]. The precision of this method depends on the quality of edge detection and the accuracy of center fitting. Rui Wang et al. developed an algorithm for OVL target center localization [26], which improves the quality and reliability of extracted contours by introducing an edge point grouping and smoothing strategy based on the Hough transform. Meanwhile, the sum of squared distances from the detected actual edge points to a customizable theoretical edge is used as the optimization objective, combined with a sub-pixel layer-by-layer traversal search strategy to improve the precision of center localization. This algorithm ultimately achieves a center localization repeatability of approximately 0.01 pixels (1σ). Although the precision of this algorithm is already at a leading level among its counterparts, its speed fails to meet the throughput requirements under advanced technology nodes.

The template matching method locates regions of interest in the original image by measuring the feature similarity between candidate regions and a reference template. The overall performance of this framework is highly dependent on two core elements: first, the appropriate definition of the similarity metric function, and second, the accuracy of the reference template information. In recent years, many researchers in the field of image processing have conducted studies on high-precision target center localization algorithms based on template matching methods [27,28,29]; however, both the precision and efficiency of these algorithms fail to meet the requirements of advanced technology nodes. Rui Wang et al. developed an algorithm [30] that introduces the facet model to extract sub-pixel edge information of the target, and proposed a composite template method to eliminate the random errors inherent in a single template. The final absolute localization accuracy achieves ±0.05 um (±0.052 pixels). Although this method improves the precision of template matching algorithms to a certain extent, it still cannot fully meet the requirements of advanced technology nodes.

In summary, both edge fitting and template matching algorithms are fundamentally devoted to extracting the absolute geometric features of targets from digital images, such as sub-pixel edges or local templates. This absolute position extraction approach based on local pixels is not only susceptible to high-frequency noise, but the complex localization strategies introduced to compensate for the lack of precision also greatly sacrifice computational efficiency. Therefore, continuing to optimize geometric feature extraction within the existing framework falls short of simultaneously meeting the stringent precision requirements and high throughput demands of advanced technology nodes.

In fact, IBO metrology tools are designed from the outset with an important physical prior condition: in order to maximize the suppression of systematic measurement errors, instrument non-idealities such as optical aberrations that are asymmetrically distributed about the OVL target center (i.e., the field of view center) are strictly controlled [11,31]. This implies that, in the final image affected by instrument non-idealities, the grayscale distributions of the target edges on both sides should be approximately symmetric about the center point.

Based on the above physical prior knowledge, this paper moves beyond the underlying logic of conventional absolute geometric center localization and proposes a novel IBO target localization algorithm based on the symmetry center. Unlike conventional methods that rely excessively on local features, the proposed algorithm directly focuses on the global symmetry characteristics of the image. It extracts the grayscale profile signal of the edge on one side of the OVL target, performs a spatial mirror flip, and subsequently uses it as a reference template to conduct a sliding cross-correlation matching computation in the edge region on the opposite side. The peak position of the correlation coefficient curve is then taken as the optimal symmetry matching point, from which the symmetry center of the target is directly and precisely determined. The algorithmic principle will be elaborated in the following sections. Simulation and experimental results presented in this paper demonstrate that the proposed algorithm meets the stringent requirements of advanced technology nodes in terms of both precision and robustness, thereby laying a solid foundation for its engineering application in practical IBO metrology tools.

## 2. Methodology

### 2.1. Algorithm Description

**Figure 2:**
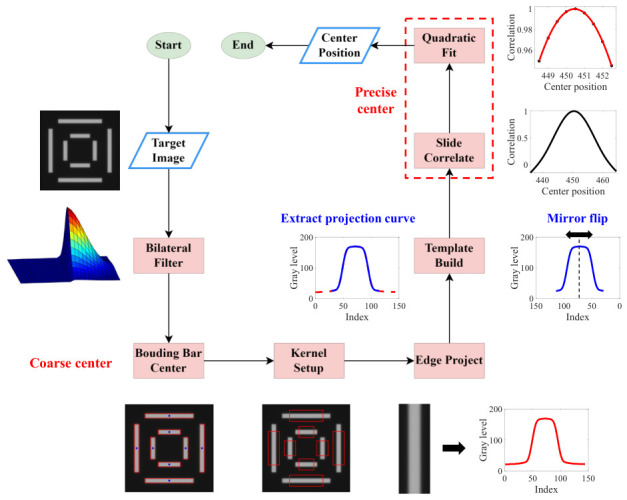
Overall algorithm flowchart.

Based on the symmetry design principle of the IBO tool, the core objective of the proposed algorithm is to precisely compute the geometric symmetry center of the OVL target measured by the system. The overall algorithm architecture not only incorporates the core correlation computation, but also integrates multiple upstream optimization strategies to ensure extremely high precision and robustness of the metrology results under complex measurement conditions. The complete processing pipeline of the algorithm is illustrated in Figure 2. In the following sections, the Bar in Bar (BiB) target, a classical OVL target in semiconductor metrology, will be used as an example to elaborate on the specific implementation steps of the algorithm. All algorithmic step figures in this section are derived from intermediate processing results of experimentally acquired OVL target images. The original image has a resolution of 1920 × 1200 pixels, from which a 1000 × 1000 region of interest (ROI) was cropped from the center for computation.

The first step is to preprocess the input raw image. Raw images carry random noise, which causes subtle fluctuations in pixel grayscale values. Even for consecutively captured images, the random noise differs from frame to frame, which degrades the repeatability precision of target center localization. Although noise was taken into account during the hardware design phase, a certain level of random noise inevitably remains in the system, necessitating that the algorithm suppresses random noise in the image as much as possible during the preprocessing step. During noise suppression, it is equally important to preserve the edge information of the target without alteration, since the center position of the target is indirectly derived from the edge information; any modification to the edge information will render the computed center position inaccurate. Conventional linear filters, such as Gaussian filters, consider only the spatial distance between pixels when smoothing noise, which inevitably leads to edge blurring. In contrast, the bilateral filter integrates both spatial proximity and grayscale similarity of pixels, thereby achieving excellent edge-preserving denoising performance [32,33]. Taking all factors into consideration, the bilateral filter is selected for random noise suppression.

For an arbitrary point of the pixel *p* in the image *I*, the output of bilateral filter, denoted $BF{\left[I\right]}_{p}$, is defined as the weighted average of its neighboring pixels *q*. The mathematical expression is as follows:

(1) $$BF{\left[I\right]}_{p}=\frac{1}{W_{p}}\sum_{q\in S}G_{\sigma_{s}}\left(||p-q||\right)G_{\sigma_{r}}\left(I_{p}-I_{q}\right)I_{q},$$

where $W_{p}$ is the normalization factor used to ensure that the total weight is equal to 1:

(2) $$W_{p}=\sum_{q\in S}G_{\sigma_{s}}\left(||p-q||\right)G_{\sigma_{r}}\left(I_{p}-I_{q}\right).$$

**Figure 3:**
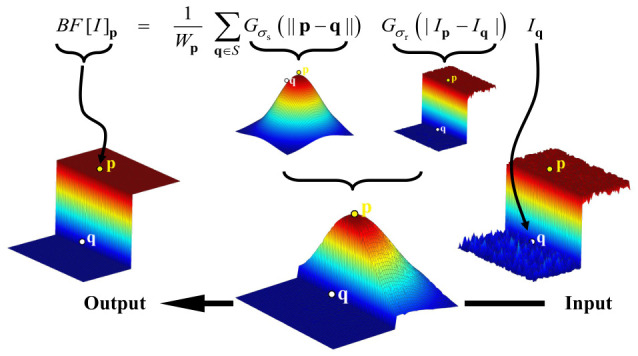
Bilateral filter on a height field.

In the mathematical model described above, the equivalent filtering weight of the bilateral filter is modulated by the product of two Gaussian kernels: the spatial domain kernel and the range domain kernel. Specifically, the spatial Gaussian weight $G_{\sigma_{s}}$ depends on the spatial Euclidean distance between pixels; the weight assigned to neighboring pixels decays exponentially as their distance from the central pixel increases, thereby defining the spatial smoothing scale of the filter. Conversely, the range Gaussian weight $G_{\sigma_{r}}$ relies on the intensity similarity between pixels, aiming to penalize the contribution weights of neighboring pixels that exhibit significant intensity deviations from central pixel $I_{p}$. This dual kernel modulation mechanism ensures that the filter effectively smooths spatial noise while successfully blocking filtering response across edge regions. Figure 3 provides a visual demonstration of the principle of bilateral filter.

**Figure 4:**
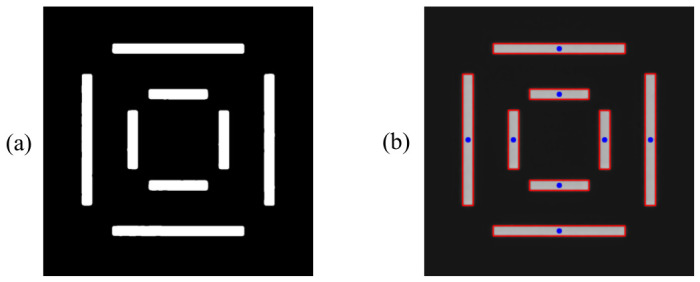
Coarse localization result. (**a**) Binarization result; (**b**) the localization result of the center of the bar’s circumscribed rectangle.

The second step is to perform coarse localization on the denoised image, where the localization targets are the center positions of each rectangular bar of the BiB target. This step serves as a preparation for the subsequent fine localization. To achieve this goal, the proposed algorithm employs a method based on connected component statistical analysis to extract the geometric center of the feature targets. Since the preceding bilateral filtering has effectively smoothed the background noise while highly preserving the edge information, the algorithm first applies adaptive thresholding (Otsu’s method) to convert the denoised grayscale image into a binary mask (Figure 4a), thereby separating the individual bars of the BiB target from the background region. Subsequently, connected component statistical analysis is applied to globally parse the binary image, simultaneously extracting key morphological features of each connected region, including the area and bounding rectangle.

Considering that minor defect spots or process-induced spurious pattern features may remain in the actual measurement field of view, the proposed algorithm further incorporates geometric prior constraints of the target. By setting specific area thresholds, all extracted connected components are filtered based on their features, precisely eliminating interference components and thereby locking onto the bars belonging to the outer and inner layers. It is worth noting that when extracting the center coordinates of each bar, the proposed algorithm does not directly adopt the sub-pixel centroid computed by the connected component analysis. Instead, the center point inferred from the geometric boundaries of the bounding rectangle is selected, because the sub-pixel centroid is highly susceptible to edge variations introduced by binarization, whereas the bounding rectangle center is considerably more robust to such variations. Furthermore, the 0.5 pixel localization resolution provided by the bounding rectangle center fully satisfies the requirements of the coarse localization stage. The bounding rectangles of all finally extracted bars are shown as red rectangles in Figure 4b, with the center positions indicated by solid blue dots.

**Figure 5:**
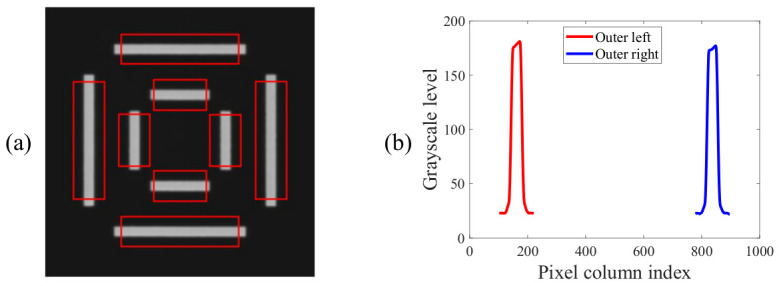
(**a**) Search kernel setup; (**b**) grayscale level projection.

The third step is to set a search window for each bar using the bar center point as an anchor, as shown by the red rectangles in Figure 5a. When setting the search window, the corners of the bar should be avoided as much as possible, since corner regions tend to exhibit more fabrication defects. It is sufficient to capture only 70–90% of the total bar length. The edge information within the search window is then extracted as a grayscale projection profile. Taking the left bar of the outer target as an example, this is achieved by computing the mean grayscale value across all rows of the sub-image within the red window, which is mathematically equivalent to a row-wise averaging operation, effectively suppressing residual random noise in the image. Figure 5b shows the grayscale projection profiles of the left and right bars of the outer target.

**Figure 6:**
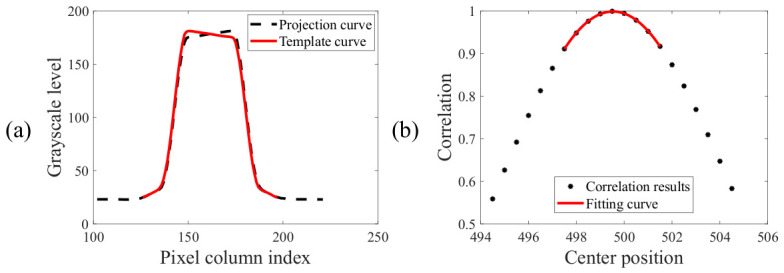
(**a**) Extract and mirror the template curve; (**b**) sliding calculation of correlation coefficient.

The fourth and final step is to compute the position of the symmetry center of the BiB target. Taking the computation of the X direction center position of the outer target as an example, a segment is first cropped from the central region of the grayscale projection profile of the left bar of the outer target and spatially mirrored to serve as the template, as shown by the red solid line in Figure 6a. The template is then slid across the grayscale projection profile of the right bar of the outer target from left to right, computing the correlation at each position. The correlation is calculated based on the mathematical expression of the Pearson correlation coefficient (Equation (3)).

(3) $$r=\frac{\sum_{i=1}^{n}\left(x_{i}-\bar{x}\right)\left(y_{i}-\bar{y}\right)}{\sqrt{\sum_{i=1}^{n}{\left(x_{i}-\bar{x}\right)}^{2}}\sqrt{\sum_{i=1}^{n}{\left(y_{i}-\bar{y}\right)}^{2}}},$$

where $\bar{x}$ and $\bar{y}$ denote the mean values of the signal and the template within the matching window, respectively. The center position corresponding to each correlation coefficient in Figure 6b is obtained by computing the average of the center pixel position of the template and center pixel position of the signal within a matching window. The position of the peak of the correlation coefficient curve corresponds to the point of highest bilateral edge overlap in physical terms, i.e., the symmetry center. To improve computational precision, a second-order polynomial fitting is performed on several discrete points in the vicinity of the maximum value of the correlation coefficient, as shown in Figure 6b. The final center position corresponds to the location of the maximum of the fitted curve, and the maximum correlation coefficient is calculated to be 0.9993.

The process quality of the target and the performance of measurement tool are the two most critical factors influencing the correlation coefficient. The test target in Figure 6b is a standard OVL target with good process quality, possessing a relatively symmetric structure. The IBO measurement tool also demonstrates excellent performance, with all optical aberrations and other factors that could cause asymmetric imaging of the target being well-controlled and their effects considered negligible. Consequently, the calculated correlation coefficient curve is relatively symmetric, with a maximum value close to 1.0. Under the premise of a high-performance IBO measurement tool, we have tested OVL targets from different process conditions and found that targets with excellent process quality can achieve a maximum correlation coefficient above 0.99, those with good process quality above 0.95, and those with poor process quality above 0.80.

Furthermore, in practical production line applications, if the measurement throughput and underlying computational resources permit, the edge on opposite side can be additionally extracted as a reference template to perform reverse symmetric matching. By taking the arithmetic mean of two center point coordinates derived from the bidirectional matching, the random truncation errors introduced by single-sided template cropping can be effectively suppressed, further enhancing the measurement precision of the algorithm.

The proposed algorithm elegantly transforms the symmetry design of the optical system into a mathematical correlation optimization problem. This strategy, based on global feature matching rather than local absolute gradient extraction, not only ensures excellent robustness of the algorithm when dealing with low-quality targets, but also significantly reduces computational complexity while maintaining measurement precision.

It should be noted that the algorithm proposed in this paper assumes that the centers of symmetry in the X and Y directions of the image coincide, which requires the axis of symmetry of the target to be aligned with the row and column directions of the camera sensor. The wafer stage mounted on our measurement tool is equipped with a precision rotational degree of freedom, and during the installation and calibration phase, the angular deviation between the axis of symmetry of the target and the row/column directions of the camera sensor was adjusted to within $0.1^{\circ }$, at which point the effect of the residual angular deviation is negligible.

In extreme cases where significant rotational deviation exists between the outer and inner targets due to process-related reasons, computing the center of symmetry by sliding correlation in the X and Y directions alone would be insufficient. In such cases, the algorithm would need to determine the center of symmetry by computing the intersection of the axes of symmetry of the outer or inner target. The axis of symmetry can be obtained by performing segmented projection of the edges and fitting the discrete symmetry center points calculated at different edge positions. However, such cases are extremely rare in practice and would impose an unacceptable computational burden that cannot meet the throughput requirements of the measurement system. A detailed discussion of this scenario is therefore beyond the scope of this paper.

### 2.2. Experimental Setup

**Figure 7:**
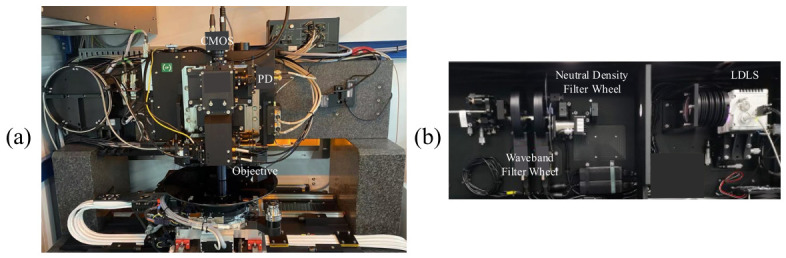
Experimental setup. (**a**) Optical measurement module; (**b**) LDLS module.

The experimental setup is shown in Figure 7. A similar IBO optical configuration has been reported in our previous work [11]. Figure 7a illustrates the optical measurement module with the BiB target as the measurement object, and Figure 7b shows the light source module equipped with LDLS, in which the filter wheel provides multiple wavelength bands for illumination selection. In this study, the selected illumination wavelength band is a white light waveband of 525–705 nm, with the illumination intensity set to ensure that the grayscale value of the target region in the captured image is approximately 180. In addition, the CMOS camera pixel size is 5.86 um, the original image resolution is 1920 × 1200 pixels, the ROI image resolution is 1000 × 1000 pixels, and the image format is single-channel 8 bit integer BMP. The objective is object-space telecentric with a magnification of 130×. The focus repeatability of autofocus subsystem is 15 nm (3σ).

The proposed algorithm is implemented in Python 3.11.5. All experimental images are processed on a computer equipped with an Intel Core i7-12700H (2.30 GHz) processor and 32 GB of RAM. Based on multiple test runs, the average execution time of the algorithm is approximately 0.07 s, in which the sliding correlation computation is performed once in the forward direction and once in the reverse direction.

According to the algorithmic principle proposed above, the computational complexity can be systematically analyzed stage by stage. Let the ROI image size be N×N pixels, the effective length of a single bar along its projection direction be *L* pixels, the effective width of a single bar in the perpendicular direction be *w* pixels, and the bilateral filter spatial radius be *r* (a small, fixed integer constant). A BiB target contains 8 bars in total: 4 outer bars and 4 inner bars. Since the inner bars are physically smaller than the outer bars, the dominant projection length is determined by the outer bars, denoted simply as *L* for brevity.

In the image preprocessing stage, the bilateral filter evaluates a weighted sum over a (2r+1)×(2r+1) spatial neighborhood for every pixel in the N×N ROI, giving a per-pixel cost of $O(r^{2})$ and an overall cost of $O(N^{2}r^{2})$. Since *r* is a fixed small constant (typically r=5–9 pixels), this reduces to $O(N^{2})$. Adaptive thresholding (Otsu’s method) requires a single pass over all pixels to construct a 256-bin intensity histogram, followed by a constant-time threshold search, so its complexity is likewise $O(N^{2})$. Connected component analysis, performed via a standard two-pass labeling algorithm, also costs $O(N^{2})$. Summing these three operations, the overall complexity of the preprocessing stage is $O(N^{2})$.

In the coarse localization stage, the number of candidate connected components *C* is bounded by a small constant determined by the target geometry. Filtering components by area threshold and computing bounding-rectangle centers therefore costs O(C)=O(1), which is negligible.

In the grayscale projection stage, computing the row-wise mean over the L×w search window of each bar costs O(Lw) per bar. With 8 bars processed independently along both X and Y directions, the combined cost of this stage is O(8·Lw)=O(Lw). Since w≪L≪N in practice, this term is dominated by the subsequent correlation stage.

In the sliding correlation computation stage, for a projection signal of length *L* and a mirror-flipped template of length kL (0<k<1), the template slides over (1−k)L+1 candidate positions. Evaluating the Pearson correlation coefficient over a window of kL elements at each position costs O(kL), so the total cost of a single sliding correlation pass is $O\;\left((1-k)L\cdot kL\right)=O\left(L^{2}\right)$. In the full algorithm, sliding correlation is carried out along both the X and Y directions, for both the outer and inner structures, and in both the forward and reverse directions to suppress single-sided truncation error, giving 2×2×2=8 independent sliding correlation passes in total. Because the number of passes is a fixed constant, the overall complexity of this stage remains $O(L^{2})$. The subsequent second-order polynomial fitting near each correlation peak operates on a constant-size neighborhood and contributes O(1) per pass.

Combining all stages, the total time complexity of the proposed algorithm is

(4) $$O\left(N^{2}+Lw+L^{2}\right)=O\left(N^{2}+L^{2}\right),$$

where the Lw term is absorbed into $L^{2}$ since w<L. In a typical measurement configuration, N=1000 and L≈150–200 pixels, so $L^{2}\ll N^{2}$ and the preprocessing stage dominates the overall runtime. Importantly, the algorithm complexity scales only with the image area $N^{2}$ and the bar projection length *L*, and is entirely independent of image content complexity, because the algorithm operates on a fixed number of structurally defined bars rather than on arbitrary image features. This property confers favorable scalability and engineering practicality for deployment in production-line IBO metrology tools.

## 3. Results and Discussion

### 3.1. Simulation

In practical IBO tools, the captured images of OVL targets are inevitably degraded by noise or blurring due to the influence of random noise and focusing errors. In this section, the performance of the proposed algorithm is tested and verified through simulations in terms of center localization accuracy, robustness against image noise, and resistance to image blurring, respectively. For the simulated images, the mean grayscale value of the target feature region is set to 180, while the mean grayscale value of the dark background is 20.

#### 3.1.1. Algorithm Localization Accuracy Evaluation

**Figure 8:**
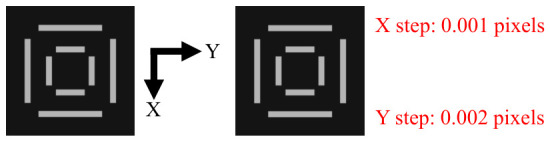
Simulate and generate target images with outer center shift.

In this paper, a series of ideal images are generated through simulation to evaluate the localization accuracy of the algorithm. The geometric dimensions of generated OVL targets are identical to those of measured targets used in the subsequent experiments (the geometric dimensions of OVL targets may vary slightly for different fabrication processes). The geometric dimensions are labeled in Figure 1, where a = 2 um, b = 10 um, c = 17 um, d = 2 um, e = 22 um, and f = 32 um. As illustrated in Figure 8, a total of 1000 images with a resolution of 1000 × 1000 pixels are simulated. For each consecutive image, the center position of the outer target increments by 0.001 pixels in the X direction and 0.002 pixels in the Y direction relative to the previous one, while the center position of the inner target remains stationary.

To achieve high-precision step-wise simulation of target center displacement, this paper employs a sub-pixel rendering method based on analytic geometric area coverage to generate simulation images. The simulation images are generated directly at the final resolution, with image coordinates, target center positions, and rectangular boundaries all represented using double-precision floating-point numbers. It should be noted that the step sizes of 0.001 pixels and 0.002 pixels for the outer target center coordinates represent only the prescribed displacement intervals between consecutive frames in the image sequence, and do not constitute the precision limit of the geometric simulation computation itself.

During image generation, the pixel intensity of each rectangular target is determined by computing the overlap area between the ideal continuous rectangular region and each pixel cell. Let the pixel center be located at the integer coordinate (i,j), with the corresponding pixel region defined as [i−0.5,i+0.5]×[j−0.5,j+0.5]. For pixels near the rectangular boundary, the overlap lengths between the pixel region and the rectangular region in the X and Y directions are computed separately, and their product yields the area coverage ratio α. The pixel intensity is then updated according to a linear blending model:

(5) $$I_{\mathrm{new}}=\alpha I_{\mathrm{fg}}+\left(1-\alpha \right)I_{\mathrm{bg}},$$

where $I_{\mathrm{fg}}$ is the foreground intensity of the target, $I_{\mathrm{bg}}$ is the background intensity, and α∈[0,1] represents the fraction of the pixel area covered by the rectangular region. Through this approach, sub-pixel center displacements are mapped onto the image intensity distribution in the form of continuous variations in the area coverage ratio. Since the target center coordinates, rectangular boundary positions, and area coverage ratios are all computed using double-precision floating-point arithmetic, the numerical precision of the geometric representation is far higher than the displacement step intervals specified in this paper, with double-precision floating-point arithmetic providing a numerical resolution on the order of $10^{-13}$–$10^{-14}$ pixels.

**Figure 9:**
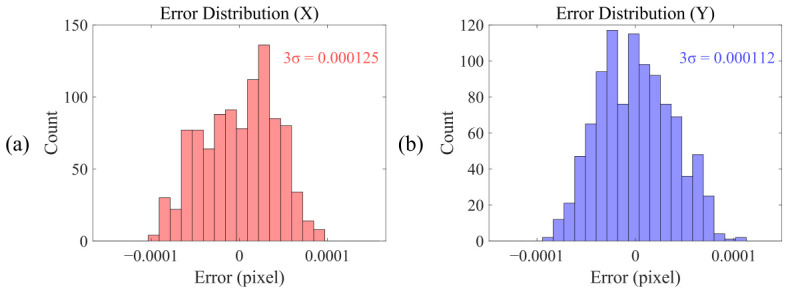
Simulation results of the central localization accuracy error of the algorithm. (**a**) X direction; (**b**) Y direction.

Upon completion of the sub-pixel rendering, the image is further convolved with a Gaussian point spread function (PSF) to simulate the optical blur of a practical imaging system. The simulation results are subsequently quantized and output as a digital image sequence following intensity mapping. To minimize the impact of simulation accuracy on the center localization accuracy of algorithm, the systematic errors caused by the simulation are eliminated by subtracting the calculated center position of the previous image from that of the subsequent image. The final center localization accuracy of the algorithm is then obtained by subtracting the corresponding step values of 0.001 pixels or 0.002 pixels from this difference. Statistical analysis was performed on the simulation results, as shown in Figure 9. Under ideal conditions, the center localization errors of the algorithm basically follow a random distribution, and the maximum error does not exceed ±0.0001 pixels.

By computing three times the standard deviation of the localization error from the simulation results, the center localization accuracy of the algorithm is obtained: 0.000125 pixels (3σ) in the X direction and 0.000112 pixels (3σ) in the Y direction. In summary, the simulation results demonstrate that the proposed algorithm achieves exceptionally high center localization accuracy under ideal conditions, fully satisfying the requirements of IBO metrology under advanced technology nodes.

#### 3.1.2. Robustness Evaluation of Algorithm Against Image Noise

**Figure 10:**
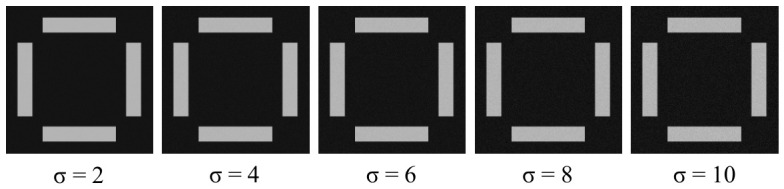
Simulation images under different noise levels.

During the actual acquisition of OVL target images, electronic non-idealities such as camera dark current introduce random noise into the captured images. To evaluate the robustness of the algorithm against noise, additive white Gaussian noise of varying magnitudes is superimposed onto the ideal target image via simulation, as shown in Figure 10. For a clearer visualization of the noise superimposition effect, only the inner region cropped from the center of the original image is shown for illustration purposes.

**Figure 11:**
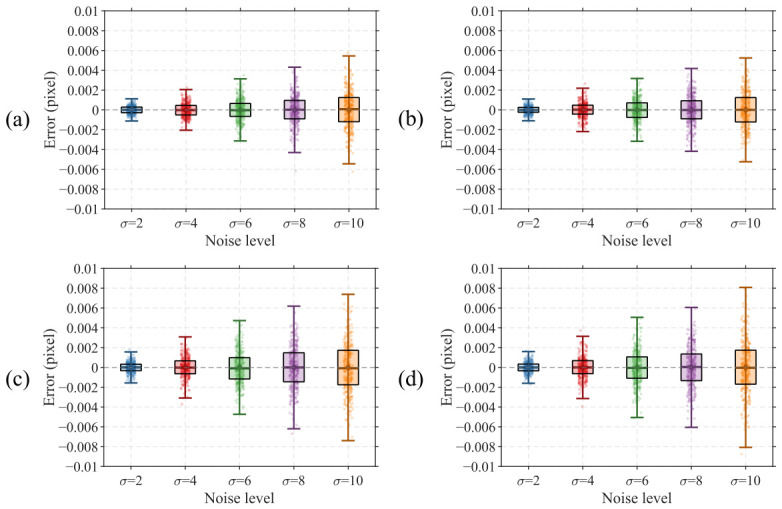
Calculation results of simulation images with different noise levels. (**a**) Outer X; (**b**) Outer Y; (**c**) Inner X; (**d**) Inner Y.

Under typical conditions, the dark background noise of experimentally acquired images does not exceed 3 grayscale levels. To thoroughly validate the algorithm performance, random noise with standard deviations of 2, 4, 6, 8, and 10 grayscale levels is superimposed onto the ideal target image, with 300 images generated for each group. The results are presented in Figure 11. In the box plot, the center line of each box represents the mean value of the center position computation results, and the height of the box represents the interquartile range covering 50% of the data distribution. The upper and lower bounds of the whiskers represent the range of mean ± 3σ, covering 99.73% of the data under a normal distribution, which is consistent with the precision evaluation convention in the semiconductor metrology field. The data points of each group are overlaid as jittered scatter points to present the complete distribution of the raw data.

As shown in Figure 11, under all noise intensity conditions, the mean localization error of the algorithm remains close to zero, and the boxes exhibit good symmetric distribution about zero, indicating that the algorithm is free from significant systematic bias and demonstrates high localization accuracy. The whiskers cover the vast majority of scatter points, verifying that the localization error approximately follows a normal distribution, which is consistent with the statistical behavior expected under random noise interference.

As the noise standard deviation increases from 2 to 10 grayscale levels, the repeatability precision of the algorithm for X direction center localization of the outer target degrades gradually from 0.00112 pixels (3σ) to 0.00545 pixels (3σ), Y direction center localization precision of the outer target degrades from 0.00110 pixels (3σ) to 0.00524 pixels (3σ), X direction center localization precision of the inner target degrades from 0.00157 pixels (3σ) to 0.00739 pixels (3σ), and Y direction center localization precision of the inner target degrades from 0.00160 pixels (3σ) to 0.00808 pixels (3σ). The statistical results indicate that the center localization repeatability precision of the inner target is slightly inferior to that of the outer target. This is attributed to the fact that the algorithm computes the grayscale projection profile of each edge by row-wise averaging, and the long-side dimension of inner bars in the BiB target is inherently smaller than that of outer bars. Consequently, fewer pixels participate in averaging computation during the grayscale projection of the inner target, resulting in less-effective noise smoothing compared to the outer target.

In summary, when images contain a certain level of noise, the proposed algorithm maintains stable and reliable localization repeatability precision. Under practical image acquisition conditions where background noise does not exceed 1 grayscale level, even higher localization repeatability precision can be achieved, fully demonstrating that the proposed algorithm exhibits strong robustness against image noise.

#### 3.1.3. Robustness Evaluation of Algorithm Against Image Blur

**Figure 12:**
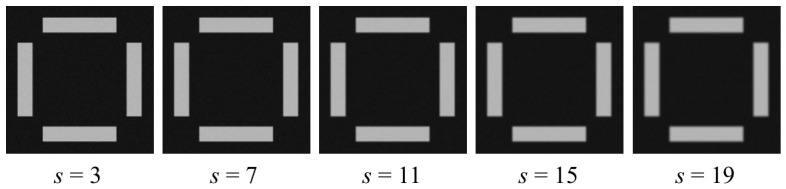
Simulation images under different degrees of blurriness.

During actual OVL target image acquisition, a certain degree of image blur is introduced due to focus errors and limited optical resolution of the system. To evaluate algorithm robustness against image blur, images with varying degrees of blur are generated via simulation, as shown in Figure 12. For clearer visualization of different blur effects, only the inner region cropped from the center of the original image is shown for illustration purposes.

**Figure 13:**
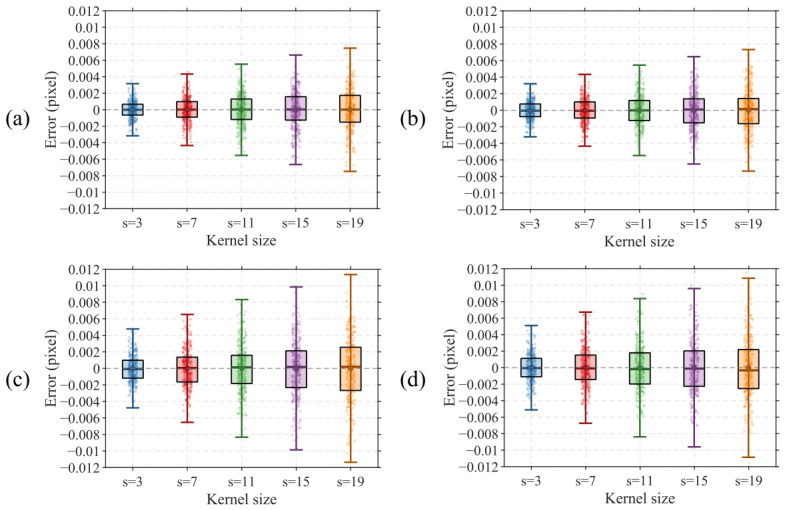
Calculation results of simulation images with different degrees of blurriness. (**a**) Outer X; (**b**) Outer Y; (**c**) Inner X; (**d**) Inner Y.

Gaussian blur with convolution kernel sizes of 3, 7, 11, 15, and 19 is applied via simulation to the image groups with a noise standard deviation of 6 from Section 3.1.2, and the results are presented in Figure 13. As the degree of blur in the simulated images increases progressively, the X direction center localization repeatability precision of the outer target degrades from 0.00316 pixels (3σ) to 0.00747 pixels (3σ), the Y direction center localization repeatability precision of the outer target degrades from 0.00320 pixels (3σ) to 0.00734 pixels (3σ), the X direction center localization repeatability precision of the inner target degrades from 0.00477 pixels (3σ) to 0.0114 pixels (3σ), and the Y direction center localization repeatability precision of the inner target degrades from 0.00511 pixels (3σ) to 0.0109 pixels (3σ).

As indicated by the above results, localization repeatability precision of the algorithm degrades gradually as image blur increases, yet overall remains at a high precision level. Furthermore, consistent with the noise robustness simulation results, localization repeatability precision of the inner target is slightly inferior to that of the outer target, which is likewise attributed to the smaller dimensions of inner bars—fewer pixels participate in grayscale projection averaging, resulting in relatively weaker suppression of blur effects. Considering that the actual degree of image blur in the system approximately corresponds to the simulation condition with a convolution kernel size of 11 (with actual noise being lower than the simulated noise), the localization repeatability precision of the algorithm in each direction under this condition is approximately 0.008 pixels (3σ), fully demonstrating that the proposed algorithm exhibits strong robustness against image blur and is capable of meeting the OVL measurement precision requirements in practical applications.

### 3.2. Experiments

**Figure 14:**
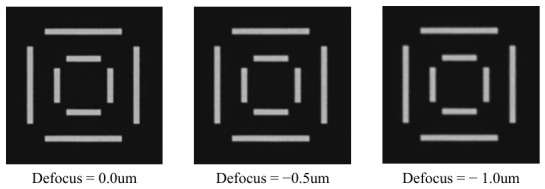
Measured images with different depth of defocus.

Experimental images of OVL targets are collected to validate various performance aspects of the algorithm, with the experimental setup described in Section 2.2. To simultaneously validate algorithm robustness against image blur and localization repeatability precision, 1500 images are captured at each of three defocus positions: 0.0 um, −0.5 um, and −1.0 um. The acquisition duration is approximately 30 s at a camera frame rate of 50 fps. OVL target images captured at different defocus positions are shown in Figure 14, where both the outer and inner structures of the measured OVL target are located on the same layer.

**Figure 15:**
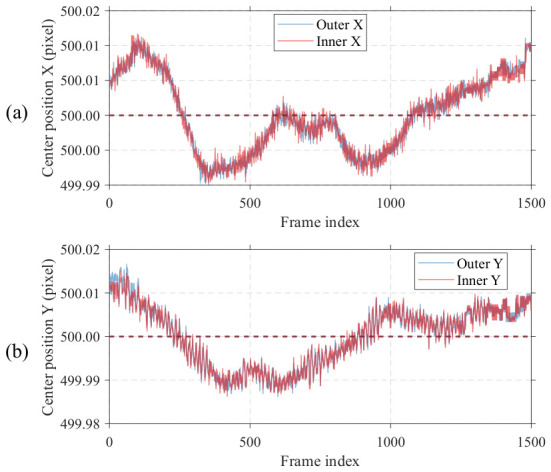
Drift of the target center position at 0 um defocus. (**a**) Center X; (**b**) Center Y.

**Figure 16:**
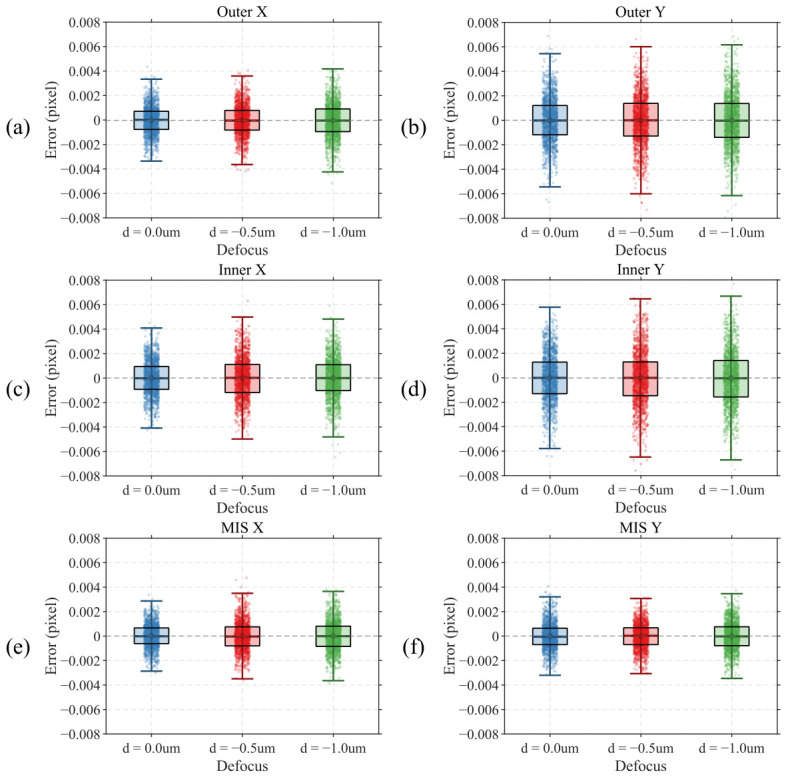
Algorithm repeatability test results. (**a**) Outer X; (**b**) Outer Y; (**c**) Inner X; (**d**) Inner Y; (**e**) MIS X; (**f**) MIS Y.

Since the continuous acquisition duration is approximately 30 s, temperature fluctuations in the test environment cause a slow nonlinear drift in the center positions of OVL targets, as shown in Figure 15. This drift trend can be eliminated by applying differential processing to the raw center position data. The differential results are shown in Figure 16a–d, where nonlinear drift is effectively suppressed. However, the differenced data still exhibits certain random fluctuations, primarily attributed to mechanical vibration interference in the test environment. Therefore, differential results cannot directly represent the actual measurement precision of the algorithm.

To address the above issue, the dual structure characteristic of the BiB target provides a more effective solution. Since the outer and inner structures are located on the same layer, the influence of both temperature drift and environmental vibration on the center positions of outer and inner structures is theoretically equivalent. Therefore, MIS obtained by computing the difference between the center positions of outer and inner structures can simultaneously eliminate the effects of temperature drift and vibration interference, without requiring differential preprocessing of the raw data. Results of directly computing MIS from the raw data are shown in Figure 16e,f.

To facilitate intuitive comparison of localization precision across experimental groups, all data in Figure 16 have been mean-subtracted; i.e., residuals obtained by subtracting the corresponding mean from each group of raw center position data or MIS data are presented, thereby eliminating the influence of absolute position offsets under different defocus conditions and focusing on data dispersion.

Figure 16 presents the statistical distribution of algorithm localization results at three defocus positions. As shown in Figure 16a–d, for the differential data of X direction and Y direction center positions of outer and inner structures, error distributions under all three defocus conditions are symmetric about zero with mean values close to zero, indicating that the algorithm is free from significant systematic bias. As defocus increases from 0.0 um to −1.0 um, both box height and whisker length increase, suggesting that increasing image blur has a certain influence on localization precision. Furthermore, center localization precision of the inner target is slightly inferior to that of the outer target. Both observations are consistent with the simulation conclusions of Section 3.1.2.

As shown in Figure 16e,f, compared with differential data, MIS-based results exhibit more concentrated scatter distributions and significantly shorter whisker lengths, confirming that MIS effectively eliminates interference from temperature drift and environmental vibration. MIS results at all three defocus positions have mean values close to zero, and the degradation in precision with increasing defocus remains limited, demonstrating that the proposed algorithm maintains stable and reliable OVL target center localization even under practical image blur conditions.

The actual autofocus error of an IBO system is typically around 15 nm. Based on the above simulation and experimental results, the influence of focus errors of this magnitude on algorithm localization precision is negligible. Therefore, MIS data acquired at a defocus position of 0.0 um is adopted as the evaluation benchmark for the final localization repeatability precision of the algorithm. Under this condition, localization repeatability precision (3σ) of the algorithm is 0.00287 pixels in the X direction and 0.00320 pixels in the Y direction.

Based on the optical magnification of the imaging system (130×) and the physical pixel size of the camera (5.86 um), image-space precision can be converted to object-space precision using the following equation:

(6) $$\delta_{\mathrm{obj}}=\delta_{\mathrm{img}}\times \frac{p}{M},$$

where $\delta_{\mathrm{img}}$ is the image-side precision (pixels), *p* is the physical pixel size, and *M* is the magnification of the optical system. After conversion, the localization repeatability of the algorithm on the object side is 0.129 nm (3σ) in the X direction and 0.144 nm (3σ) in the Y direction, which fully satisfies the OVL measurement precision requirements for advanced process nodes.

## 4. Conclusions

This paper proposes an OVL target center localization algorithm based on symmetry center matching, addressing the stringent requirements of advanced semiconductor manufacturing process nodes for IBO OVL metrology precision and throughput. Taking the symmetry design of the IBO optical system as a physical prior, the algorithm reformulates the target center localization problem as a global correlation optimization problem. By extracting the grayscale projection profile of a single-sided edge and performing mirror-flipped sliding cross-correlation matching, the symmetry center position of the target is directly computed, fundamentally circumventing the excessive dependence of conventional absolute geometric feature extraction methods on local pixel information.

Simulation results demonstrate that center localization accuracy of the algorithm is better than 0.00013 pixels (3σ). Under stringent simulation conditions far exceeding actual noise and blur levels, localization repeatability precision remains within 0.012 pixels (3σ), exhibiting excellent robustness. In experimental validation, object-space localization repeatability precision of the algorithm under accurate focus conditions is 0.129 nm (3σ) in the X-direction and 0.144 nm (3σ) in the Y-direction, surpassing the 0.32 nm measurement uncertainty requirement specified for advanced process nodes. Furthermore, the average single-frame processing time of the algorithm is approximately 0.07 s, simultaneously satisfying the dual demands of high precision and high throughput.

In summary, the proposed algorithm demonstrates excellent overall performance in terms of precision, robustness, and computational efficiency, laying a solid theoretical and experimental foundation for its engineering deployment in practical IBO metrology tools, with significant engineering application value and broad prospects for further adoption.

## Data Availability

The datasets generated and/or analyzed during the current study are available from the corresponding author upon reasonable request.
